# A novel method quantifying caries following orthodontic treatment

**DOI:** 10.1038/s41598-021-00561-7

**Published:** 2021-11-01

**Authors:** Christina Erbe, Luisa Hartmann, Irene Schmidtmann, Daniela Ohlendorf, Heinrich Wehrbein

**Affiliations:** 1 Department of Orthodontics, University Medical Center of the Johannes Gutenberg-University, Augustusplatz 2, 55131 Mainz, Germany; 2 Institute for Medical Biostatistics, Epidemiology and Informatics (IMBEI), University Medical Centre of the Johannes Gutenberg-University, Mainz, Germany; 3 Institute of Occupational Medicine, Social Medicine and Environmental Medicine, Goethe-University, Frankfurt am Main, Germany

**Keywords:** Disease prevention, Preventive medicine, Oral manifestations, Imaging techniques

## Abstract

This retrospective pilot study used a newly developed evaluation tool to assess the prevalence and incidence of White Spot Lesions (WSL) before and after multibracket appliance (MB) therapy. Digital photographs of 121 adolescent patients (63 ♂, 58 ♀) with metal brackets were analyzed retrospectively before and after MB therapy. The labial surfaces of anterior teeth, canine teeth, and premolars in the upper (UJ) and lower jaws (LJ) were evaluated using the Enamel Decalcification Index (EDI) by Banks and Richmond (Eur J Orthod, 16(1):19–25, 1994, levels 0–3) and a specially developed digitally scaled graticule with concentric circles to quantify the extent of WSL (in %). The statistical data analysis was based on crosstabulations and logistic regression. Before MB, 69.4% of the patients presented at least one WSL and 97.5% after, an increase of 28.1%. Before MB, 18.4% of the tooth surfaces (TS) showed an EDI level of 1–3. After MB, 51.8% of the TS featured WSL. 18.2% of the TS showed a WSL to the extent of ≥ 20–100% before and 52.3% after MB. The incidence in the UJ (71–79%) as well as the LJ (64–76%) was highest for the first and second premolars and lowest for LJ incisors (22–35%). The probability for developing a new distal WSL is higher than developing gingival, mesial or occlusal WSL. Labial MB therapy drastically increases the risk of developing WSL. We verified a concise quantification of the extent of labial WSL with the evaluation index.

## Introduction

The Fourth German Oral Health Study (*Deutsche Mundgesundheitsstudie* DMS IV)^1^, conducted in 2005, showed that for 45% of children (age 12) and for 58% of teenagers (age 15), orthodontic treatment had already been initiated or planned. It can be assumed that around 80% of these patients receive a permanent orthodontic appliance. The use of multibracket appliances (MB) increases plaque retention that, over the course of treatment by way of demineralization, may cause decay^2–5^. Several studies show that the occurrence of plaque is considerably higher in patients with labial MB compared to coeval patients without MB^6,7^. Despite intense efforts to educate, instruct, and motivate patients regarding effective oral hygiene procedures, demineralization, so-called white spot lesions (WSL, incipient lesion, active initial lesion), are a common clinical problem with great significance. The literature specifies that occurrence of WSL is common during fixed orthodontic treatment with an incidence and prevalence rate of 45.8% and 68.4%, respectively^8^. This large variation is due to differences with respect to the number of examined tooth types, analytical methods, age at the beginning and end of treatment, duration of treatments, materials used, ethnic origins, and patient selection^9^ (Table 1).

**Table 1:** Literature overview of the corresponding parameters for analyzing the prevalence and the incidence of WSL after MB treatment.

Author (year)	Number of patients	Teeth	Tooth surfaces	Used index	Prevalence	Incidence	Diagnosis
Lucchese et al. (2012)	191	UJ and LJ anterior teeth, posterior teeth (16–26,36–46)	4584	Gorelick et al.	40–43%	n.s	Visual inspection
Enaia et al. (2011)	400	UJ anterior teeth (12–22)	1600	Gorelick et al.	73.5%	60.9%	Intraoral photography
Lovrov et al. (2007)	53	UJ and LJ anterior and posterior teeth (17–27,37–47)	1414	Gorelick et al.	26.4%	24.9%	Intraoral photography
Pancherz and Mühlich (1997)	108	UJ anterior teeth and premolars (15–25)	794	Modif. Gorelick	62.0%	49.6%	Intraoral photography
Banks and Richmond (1994)	80	UJ and LJ anterior and posterior teeth (15–25 a. 35–45)	4728	EDI	n.s	74%	Visual inspection
Øogard et al. (1989)	51	UJ and LJ anterior and posterior teeth (16–26 a. 36–46)	1224	Modif. Gorelick	96%	n.s	Visual inspection
Årtun and Brobakken (1986)	120	UJ and LJ anterior and posterior teeth (15–25 u. 35–45)	2400	Cavity index by von der Fehr and modif. Gorelick	52.5%	n.s	Visual inspection
Mizrahi et al. (1982)	269	UJ and LJ anterior teeth and premolars	5758	Modified Index by Curzon and Spector	84%	n.s	Intraoral photography
Akin et al. (2013)	150	UJ and LJ first molar to other first molar for both jaws	n.s (3600)	Gorelick et al.	65%	55%	Intraoral photograohy
Strateman and Shannon (1974)	209	n.s	n.s	n.s	n.s	2–66%	Visual inspection, intraoral photography, roentgenograms
Khalaf (2014)	45	UJ and LJ anterior and posterior teeth (16–26 a. 36–46)	1080	Modified Index by Kuhnisch et al.	n.s	42%	Visual inspection

A WSL typically develops where plaque is able to remain on the tooth surface for a certain amount of time^10^. During MB treatment predominantly observed in the area of orthodontic brackets, archwires, loops and ligatures, which aggravate oral hygiene^2,3,11–13^, mostly in the less accessible gingival part of the tooth^2^. Moreover, MB impairs the physiological self-cleaning process based on unobstructed movements of the orofacial muscular system and the salivary flow rate^14^. The use of topical fluorides can significantly reduce the risk of WSL^15^ when applied locally in various forms to reduce demineralization processes and stimulate remineralization^16,17^.

The varying ages of the study subjects lead to a diverse set of data in regards to the prevalence and incidence of WSL. Some studies concluded that the risk of developing new and more severe lesions is significantly higher in male than in female patients^8,12,18–20^ while, Gorelick et al.^10^ detected a higher incidence in females. Geiger et al., however, could not verify a significant distinction^21^.

Upon inspecting the different tooth types for the development of labial WSL, the prevalence was significantly higher on the lateral incisor of the upper jaw and for the premolars of the lower jaw^2,10,19,21–23^. Furthermore, several studies have shown that WSL develop primarily on the gingival tooth surface. Plaque retention develops particularly on the lateral incisor due to the small space between bracket and marginal gingival, which also increases the risk of demineralization^2,10,21,24^. On the upper incisor, labial WSL could already be detected four weeks after beginning MB therapy^25,26^.

The large variation of measured WSL is due to the applied analytical methods. Gorelick et al. developed the first clinical-visual research method for evaluating WSL^10^. Inaccuracies result when different examiners evaluate identical patients based on the WSL index (interexaminer reliability). Therefore, a research calibration as well as a standardized evaluation procedure is necessary for using this index.

Årtun and Brobakken modified this index to draw more precise conclusions regarding the extension of WSL^2^. The results of their research showed high inter-rater reliability. In order ro assure a precise monitoring process of the rate of demineralization, each tooth surface was inspected separately. Årtun and Brobakken’s index was further modified by Banks and Richmond to include the evaluation of tooth surfaces affected by carious lesions, which is also known as the Enamel Decalcification Index (EDI)^22^. Overall, they sum up the results of the evaluated surfaces of each tooth and each patient to determine the degree of demineralization.

Quantitative light-induced fluorescence (QLF) determined the highest WSL prevalence. Contrary to direct visualization, QLF is considered an objective diagnostic tool, albeit a substantially more labor and cost intensive one, when compared to direct visualization. The disadvantage of this method is that alongside demineralizations, calculus, plaque, and discoloration also fluoresce differently than healthy tooth enamel^27^. According to Chapman et al., the use of imaging software is a valid method to evaluate digital photos and to assess the incidence and severity of WSL on the front teeth and premolars^15^. Kavvadia et al. stated that fluorescence devices for the chairside diagnosis and quantification of WSLs in orthodontic patients perform moderately, and have no greater advantage than the visual diagnostic methods^28^.

Therefore, this study was conducted to evaluate the prevalence and incidence of WSL before and after labial MB therapy. The intensity and extent of WSL were determined using the well-known Enamel Decalcification Index (EDI) by Banks and Richmond and extended by a newly developed digital evaluation tool, a graticule with concentric circles.

## Methods

We examined the medical records of all patients who had been treated with MB appliances in the upper jaw (UJ) and lower jaw (LJ) and had completed their treatment within the last 10 years. The following inclusion criteria had to be fulfilled: (1) no previous treatment with a multibracket appliance, (2) no crowns/veneers, restoratives, or structural changes in the enamel on the buccal surfaces of the teeth to be examined, neither before nor after MB treatment, (3) a conventional metal bracket Discovery® with 0.22 Slot (Dentaurum, Ispringen, Germany) was pasted on all anterior teeth, canine teeth, and premolars in the upper and lower jaw, (4) MB treatment time had to be at least 1 year, (5) availability of the complete documentation records (intraoral photographs) of the time prior to and after MB therapy. Unfortunately, no information on subjects’ socioeconomic status, frequency of consulting a general dentist, or water fluoridation in areas of previous residency was available.

At the beginning of MB treatment, a dental hygienist instructed all patients with the same specifications for their daily at home oral care. No professional was involved in the patient’s day-to-day tooth cleaning process. The orthodontist recommended that the patients use their toothbrushes and interdental brushes after meals, 2–3 times daily, to use fluoride toothpaste and to use mouth rinse. In addition, the orthodontist advised them to use a fluoride gel once a week after brushing their teeth at night (Elmex Gel, GABA International, Therwil, Switzerland).

One examiner (L.H.), under the direct supervision of the principal investigator (C.E.) analyzed intraoral digital photographs of 313 patients, treated with a labial MB appliance in the upper and lower jaw. Patients over the age of 18 were excluded from the study, leading to the exclusion of 76 (24.3%) patients from the study. There were no photographs taken directly before the adhesive bonding of the bracket of 47 (15%) other patients. After debonding the brackets, pictures of 54 patients (17.3%) were unavailable. Due to the poor quality of photographic images, 5 patients (1.6%) could not be analyzed adequately. Three cases (0.96%) were not evaluated as these patients were treated in combination with a fixed functional Herbst appliance for Class II correction.

In the end, a sample population of 121 patients (63 male, 58 female) were included in the study, adding up to n = 9680 tooth surfaces, the largest amount of tooth sufaces reported in literature^2,10–12,18,24,29–33^. At the beginning of the multibracket appliance treatment, their average age was 12.5 ± 2.2 and the average treatment time was 3.1 ± 1.4 years.

All of the analyzed intraoral photographs were taken in a standardized manner at the Department of Orthodontics, University Medical Center of the Johannes Gutenberg-University, Mainz, with a digital reflex camera with a RGB-CCD-sensor (Nikon D80, Nikon Corporation, Chiyoda, Tokio, Japan). The maximum resolution was 10.2 megapixels (corresponding to 3872 × 2592 pixels). In addition, we used a macro lens (105 mm, Sigma F2.8 DG Macro, Kawasaki, Japan) with an appropriate ring flash (Sigma electronic Flash Macro EM-140 DG, Sigma Corporation, Kawasaki, Japan).

Three standardized digital photographs (frontal, right lateral and left lateral photographs) from the time before and after MB treatment were evaluated. The four anterior teeth, canine teeth, and the first and second premolars in UJ and LJ were evaluated. Molars were excluded because bands were applied during MB treatment.

**Figure 1:**
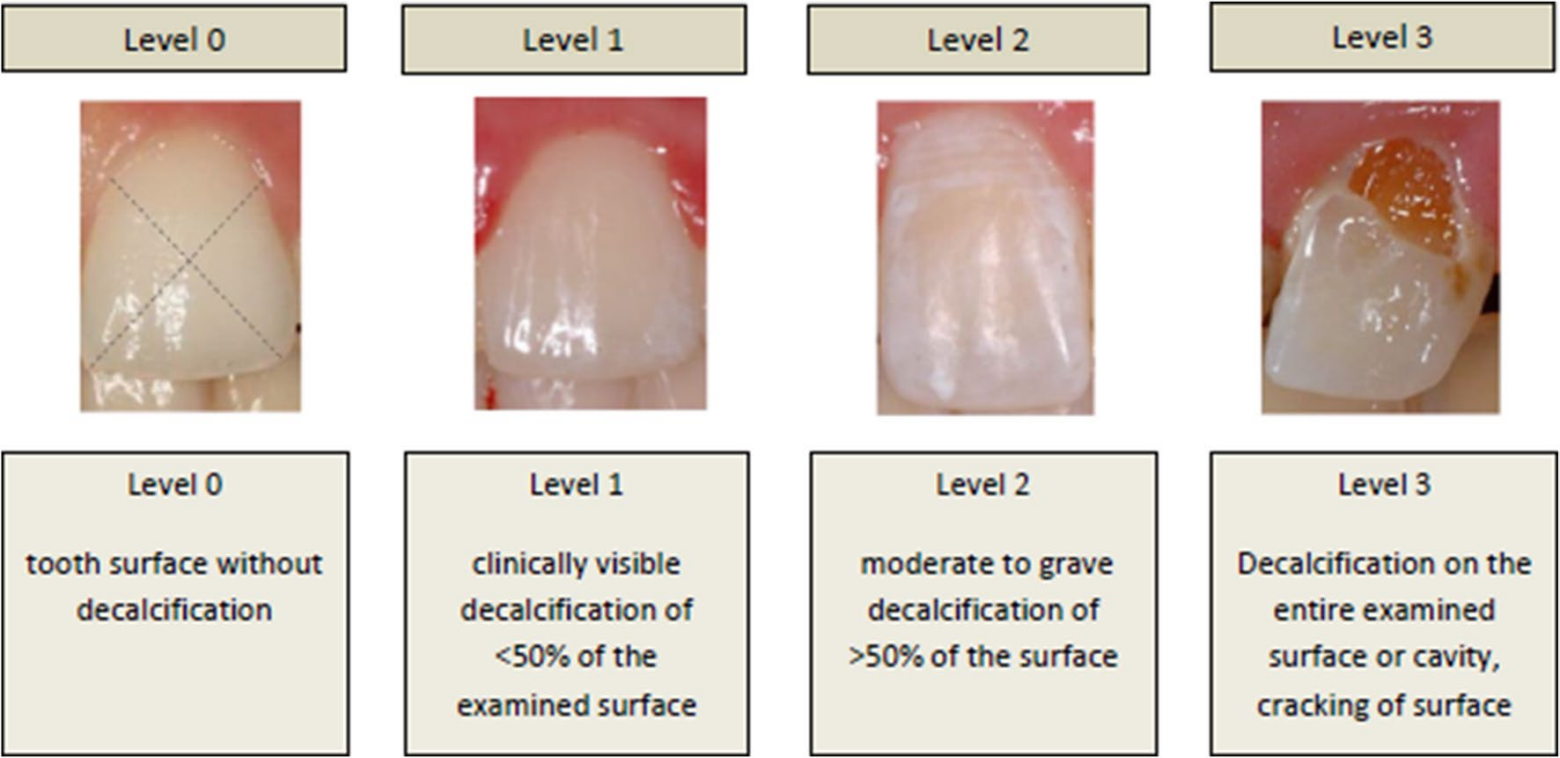
Description of the Enamel Decalcification Index (EDI) by Banks and Richmond to measure the intensity of White Spot Lesions.

The EDI by Banks and Richmond^22^ was applied to evaluate the intensity of WSL on the labial tooth surfaces before and after MB therapy (Fig. 1).

Measures were taken to avoid examiner fatigue, including limiting the photo reviewing sessions, and taking breaks after each patient.

**Figure 2:**
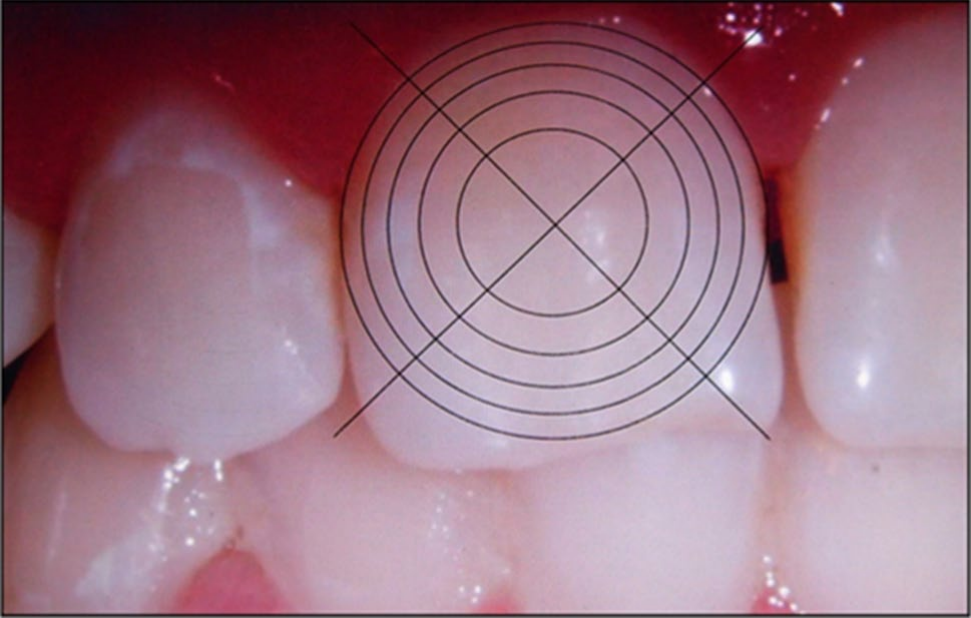
Evaluation tool with graticule and concentric circles.

Overall, the results of the analyzed surfaces were combined for each individual tooth. Since the EDI only considers the intensity of demineralization, we innovated the method to be able to additionally evaluate the extent of the lesions by preparing a digital evaluation tool. The graticule reflects the division of the EDI according to Banks, which divides the tooth surface into four areas. Each concentric corresponds to 20% of the tooth surface. This evaluation tool with graticule and concentric circles was adapted to each tooth that was subject to evaluation (Fig. 2).

The photographs were interpreted according to a standardized procedure in a dark room by one calibrated examiner on a computer (ACER Aspire 6920G, Acer Inc, Taipeh, Taiwan) with a PnP monitor and NVIDIA GeForce 9500 M GS with 1366 × 768 pixels (color depth 32 bit). The digital photographs were opened with the program “Windows Photo Gallery” (Microsoft Corporation, Redmond, USA), and each tooth subject to evaluation was digitally adapted to the size of the evaluation tool. First, pictures that were taken before brackets had been fastened were examined. The examination was conducted the the following order: tooth 11 and 12 frontally, tooth 13 to 15 laterally, and subsequently continuing with the second, third, and fourth quadrants. The first data collected corresponded to the extent (in %), whereas the second data collected corresponded to the intensity of WSL (in line with the EDI). Each picture was analyzed by one examiner.

### Statistical evaluation

The statistical evaluation of the data was conducted in collaboration with the Institute for Medical Biometry, Epidemiology, and Informatics Mainz (IMBEI) using SPSS (V.17.0.1 for Windows, IBM Corporation, Armonk, USA).

For the intra-rater reliability, the Intraclass Correlation Coefficient (ICC) was calculated. Hence, an inference could be drawn about the reproducibility within the three evaluations of the first five patients.

The examined surfaces of each tooth were summarized after the plausibility check.

In the process, the indices of intensity (Enamel Decalcification Index, EDI) were binary-recoded. 0 stands for no WSL and 1 for the existence of a WSL. Prior to and after MB treatment, the prevalence of WSL on each tooth was calculated and crosstabulations were created based on the following formulas:

$${\text{Prior }}\,{\text{prevalence}} = {\text{prior}}\,{\text{ WSL}}/{\text{total }}\,{\text{number }}\,{\text{of }}\,{\text{patients}}$$

$${\text{Subsequent }}\,{\text{prevalence}} = {\text{subsequent}}\,{\text{ WSL}}/{\text{total }}\,{\text{number}}\,{\text{ of}}\,{\text{ patients}}$$

In addition to evaluating the prevalence of WSL before and after MB treatment, the distribution of the examined indices (EDI and evaluation index respectively) was illustrated with respect to the frequency of WSL emergence per tooth.

The data collected on the intensity of demineralization (EDI index) allowed us to draw meaningful conclusions about the presence of WSL.

In order to illustrate the incidence of WSL after MB treatment, the evaluation was conducted using crosstabulations and the following formula:

$${\text{Incidence}} = \left( {{\text{number }}\,{\text{without }}\,{\text{prior }}\,{\text{WSL }}\& {\text{ number }}\,{\text{with }}\,{\text{subsequent }}\,{\text{WSL}}} \right)/{\text{number}}\,{\text{ without }}\,{\text{prior }}\,{\text{WSL}}$$

The following formula was used ro interpret the incidence. The calculation considers the upper and lower boundaries of the confidence interval.

$${p}_{\mathrm{1,2}}=\frac{2m+{u}^{2}\pm u \sqrt{{u}^{2}+4m(1-p)}}{2\left(n+{u}^{2}\right)}$$

The incidence lies within the calculated range and has a probability of p = 0.95. Additionally, the examined indices (EDI and evaluation index) were illustrated with respect to the frequency of re-emergence after MB treatment.

A sample size calculation was performed in collaboration with a statistician of the IMBEI.

The binary logistic regression analysis was conducted to statistically evaluate significant influence factors, where all tooth psotions were combined to create the model. Therefore only one (arbitrary) refrence tooth was chosen. Gender, duration of treatment, tooth type, and tooth surface were defined as variables to be examined. We decided to use tooth 11 as reference, a second reference was neither necessary nor possible. We considered data p < 005 as statistically significant. In total, we devised three analytical models: 1. WSL prior to MB treatment, 2. WSL after MB treatment, and 3. incidence of WSL.

### Ethics approval and consent to participate

All investigations and procedures were conducted according to the principles expressed in the Declaration of Helsinki. Ethical approval for the retrospective study was not required. The Ethics Committee letter, supporting that statement, is added separately. No experiments involving human participants (or their tissue) were conducted. The analysis was performed only by analyzing anonymized digital photographs of the patients’ teeth.

## Results

The intra-rater reliability of evaluating the digital photographs resulted in an ICC of 0.99 for the EDI index and of 0.96 for the evaluation index.

A patient was considered to be affected if the surface of one tooth showed a WSL. We examined 121 patients with 20 teeth each and 4 tooth surfaces (in total n = 9680 tooth surfaces).

In total, 69.4% of patients (65% male and 74% female) had a WSL prior to MB therapy. After MB therapy, 97.5% of patients (98.4% male and 97% female) presented at least one WSL. The incidence of patients affected by WSL amounted to 28.1%.

**Figure 3:**
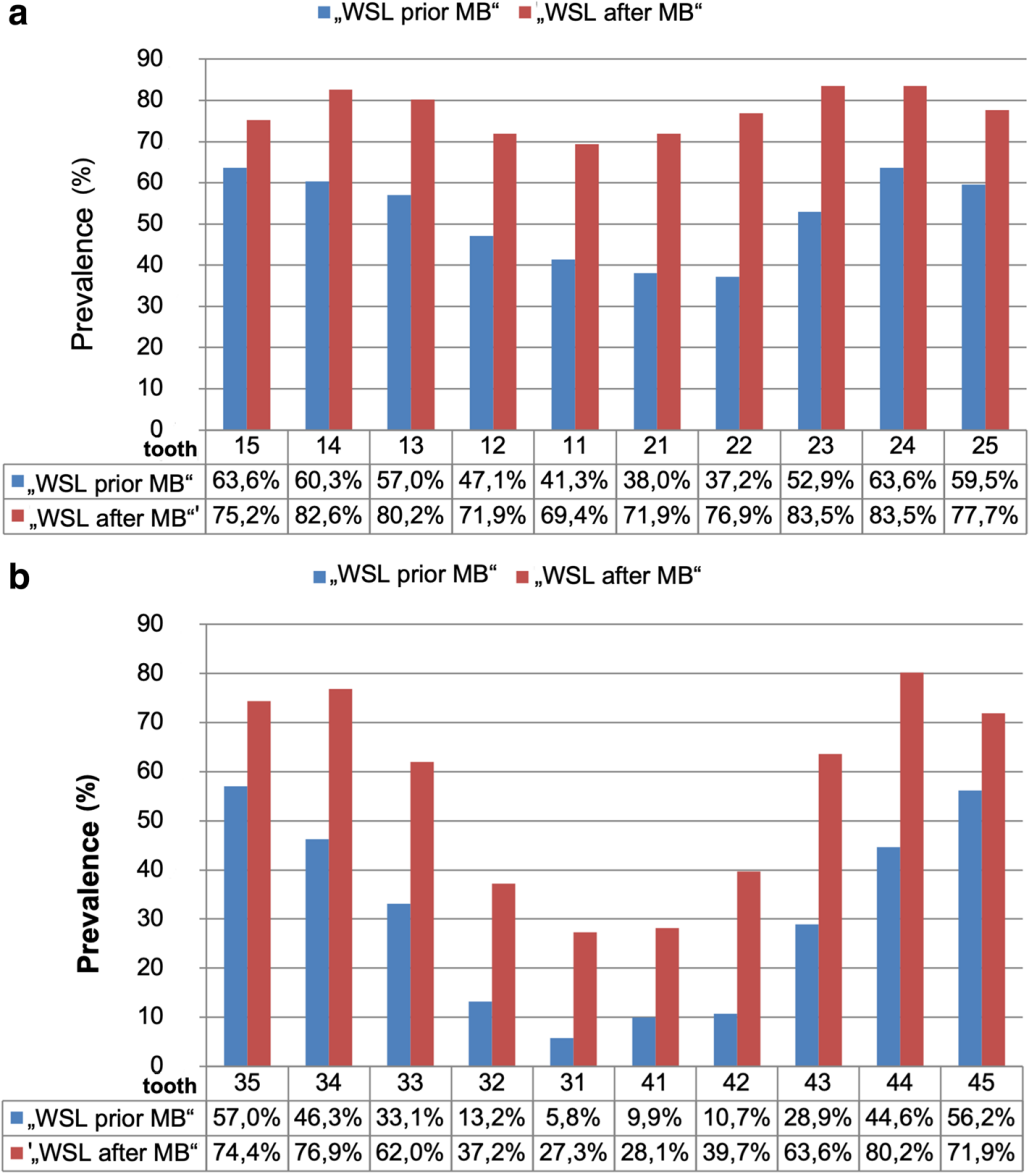
(**a**, **b**) Bar diagrams describing the prevalence of WSL of upper teeth (**a**) and lower teeth (**b**). The x-axis divides the examined teeth according to the time of their examination (WSL prior to MB; WSL after MB). The y-axis displays the percentage that corresponds to the occurrence of WSL; only the positive results (WSL = 1) were included.

The right and left posterior teeth were more often affected by WSL before MB therapy than canine and anterior teeth in the upper jaw. The second premolar on the right (63.6%) and the first premolar on the left (63.6%) were most often affected by WSL prior to treatment, and after orthodontic treatment, the most commonly affected teeth in the upper jaw were the premolars and canine teeth (75.2–83.5%) (Fig. 3a).

Similarly to the upper jaw, the lower right and left posterior teeth were more often affected by WSL before treatment than the canine and anterior teeth.

**Figure 4:**
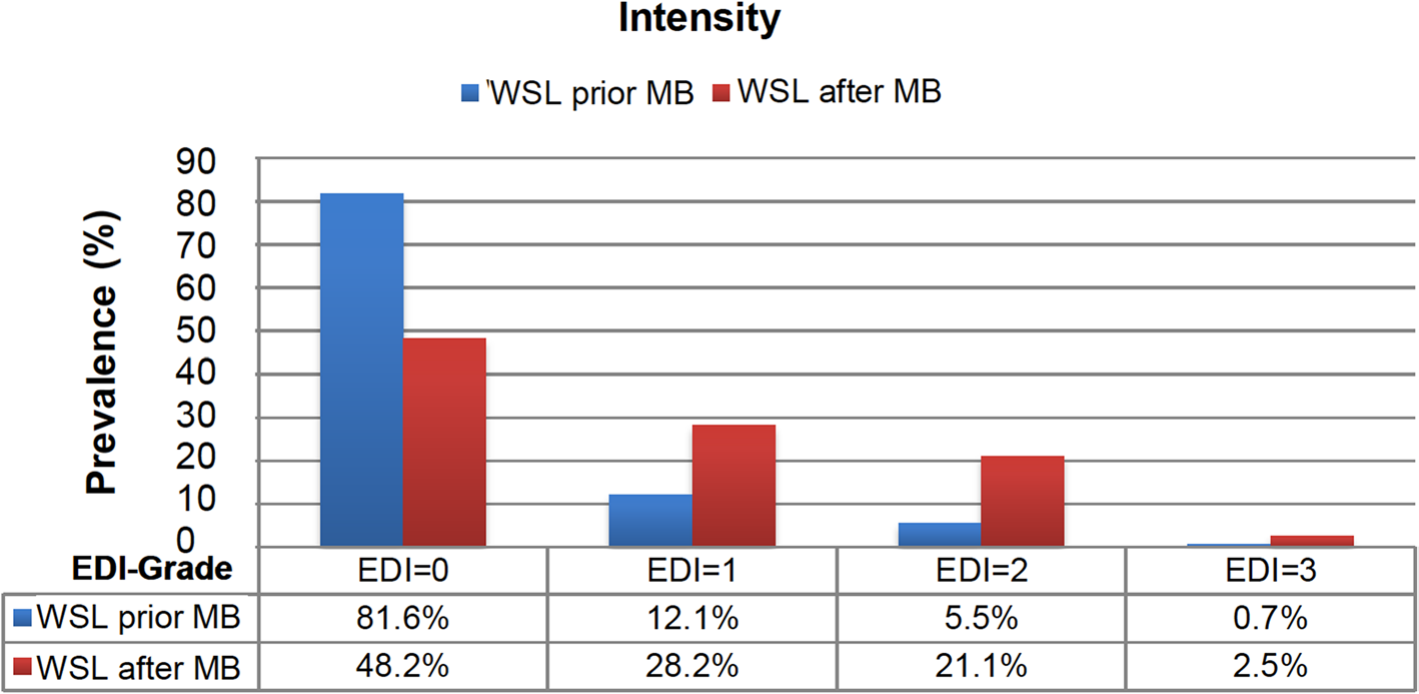
Bar diagram describing the WSL prevalence distributed by percentage of intensity (with Enamel Decalicification Index, EDI). The x-axis shows EDI levels 0–3 and their percentage of the WSL evaluation prior to and after MB. The y-axis specifies the prevalence in %.

**Figure 5:**
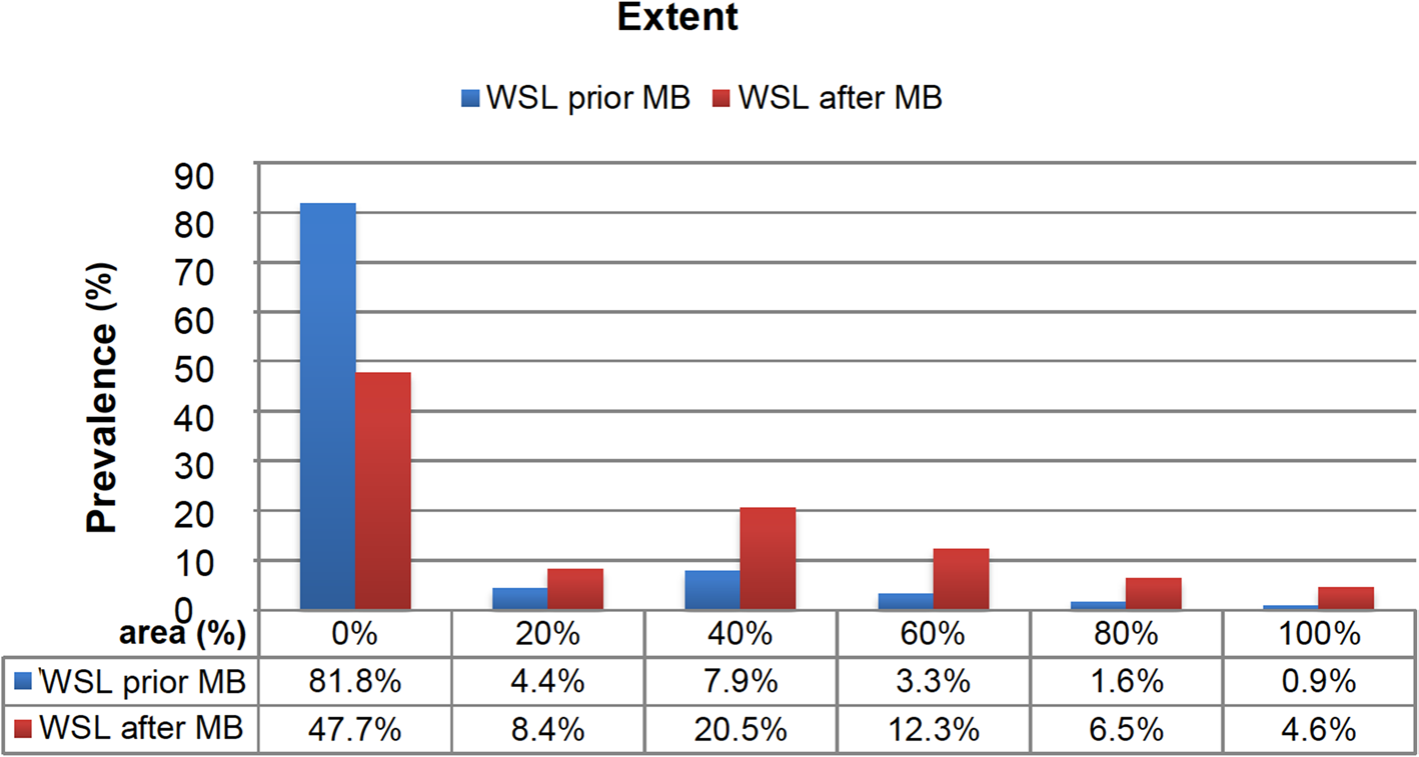
Percentaged distribution of the evaluation index according to the occurrence of WSL. The x-axis of the bar diagram shows the evaluation index (0–100%) and its distribution by percentage upon evaluating WSL, prior to and after MB respectively. The y-axis shows the prevalence in %.

Both second premolars were most often affected by at least one WSL (56.2–57%). After completing MB treatment, most of the WSL appeared, as in the upper jaw, on the right and left premolars and also canine teeth (Fig. 3b). Prior to MB therapy, 81.6% of all tooth surfaces did not show a WSL, and 18.4% showed mild to grave WSL. After MB therapy, we detected WSL with an EDI level of 1–3 on more than half of the tooth surfaces (51.8%). The prevalence of WSL intensity decreased along with an increasing EDI level prior to and after MB therapy (Fig. 4). Before MB therapy 18.2% of the tooth surfaces presented a WSL with an extent of ≥ 20–100%, which increased to 52.3% after MB treatment. The majority (40.8%) of newly developed WSL concerned 40% of the tooth surfaces. After MB treatment, an increase of the extent could be found in all areas in a range between 20 and 100% with a peak at 40% (Fig. 5). The incidence – the number of new cases – was highest in the upper canine teeth and first premolars (71–79%), followed by lateral incisors and second premolars (59–63%). In the LJ, the incidence was highest in the second and first premolars (64–76%) and lowest in the incisors (22–35%) (Table 2).

**Table 2:** Incidence of every single tooth and the corresponding confidence intervals.

Tooth	Confidence interval 95%		Incidence (%)
	Lower boundary	Upper boundary	
11	0.3668	0.5931	48
12	0.4869	0.7194	61
13	0.5975	0.8323	73
14	0.5682	0.8176	71
15	0.4441	0.7231	59
21	0.4345	0.6543	55
22	0.5192	0.7312	63
23	0.6671	0.8753	79
24	0.5815	0.8365	73
25	0.4725	0.7357	61
31	0.1757	0.3321	25
32	0.2165	0.3885	30
33	0.4233	0.6357	53
34	0.6042	0.8171	72
35	0.6179	0.8477	75
41	0.1527	0.3068	22
42	0.2683	0.4456	35
43	0.4990	0.7014	60
44	0.6467	0.8473	76
45	0.5069	0.7570	64

**Figure 6:**
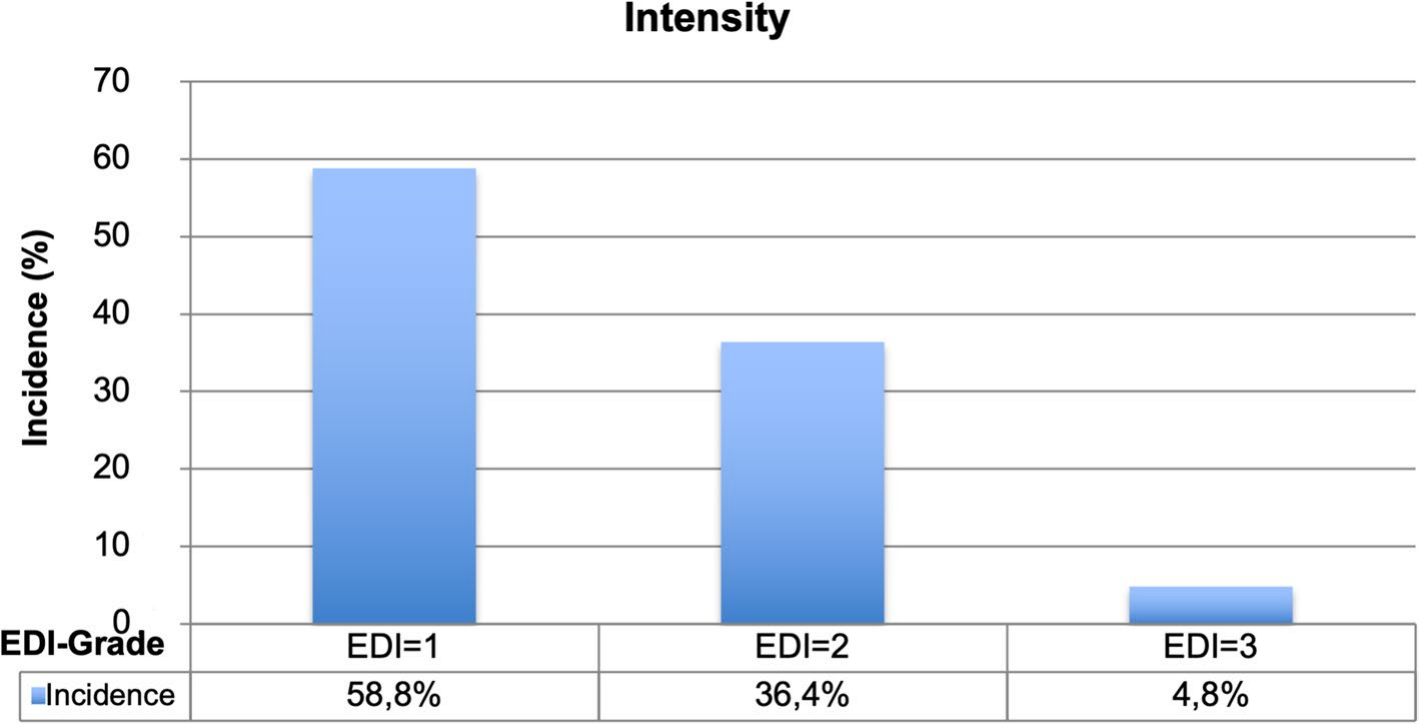
Incidence distribution based on intensity (EDI).

In order to accurately depict the incidence according to extend and intensity of WSL after MB treatment, only the tooth surface of patients who did not present a WSL at the beginning of the study were examined. 58.8% of the newly affected tooth surfaces showed enamel demineralizations on less than 50% of the tooth’s surfaces (EDI = 1). 36.4% exhibited enamel demineralizations on more than 50% of the tooth’s surfaces (EDI = 2). 4.8% of the incidences featured demineralizations on 100% of the tooth’s surfaces (EDI = 3) (Fig. 6).

**Figure 7:**
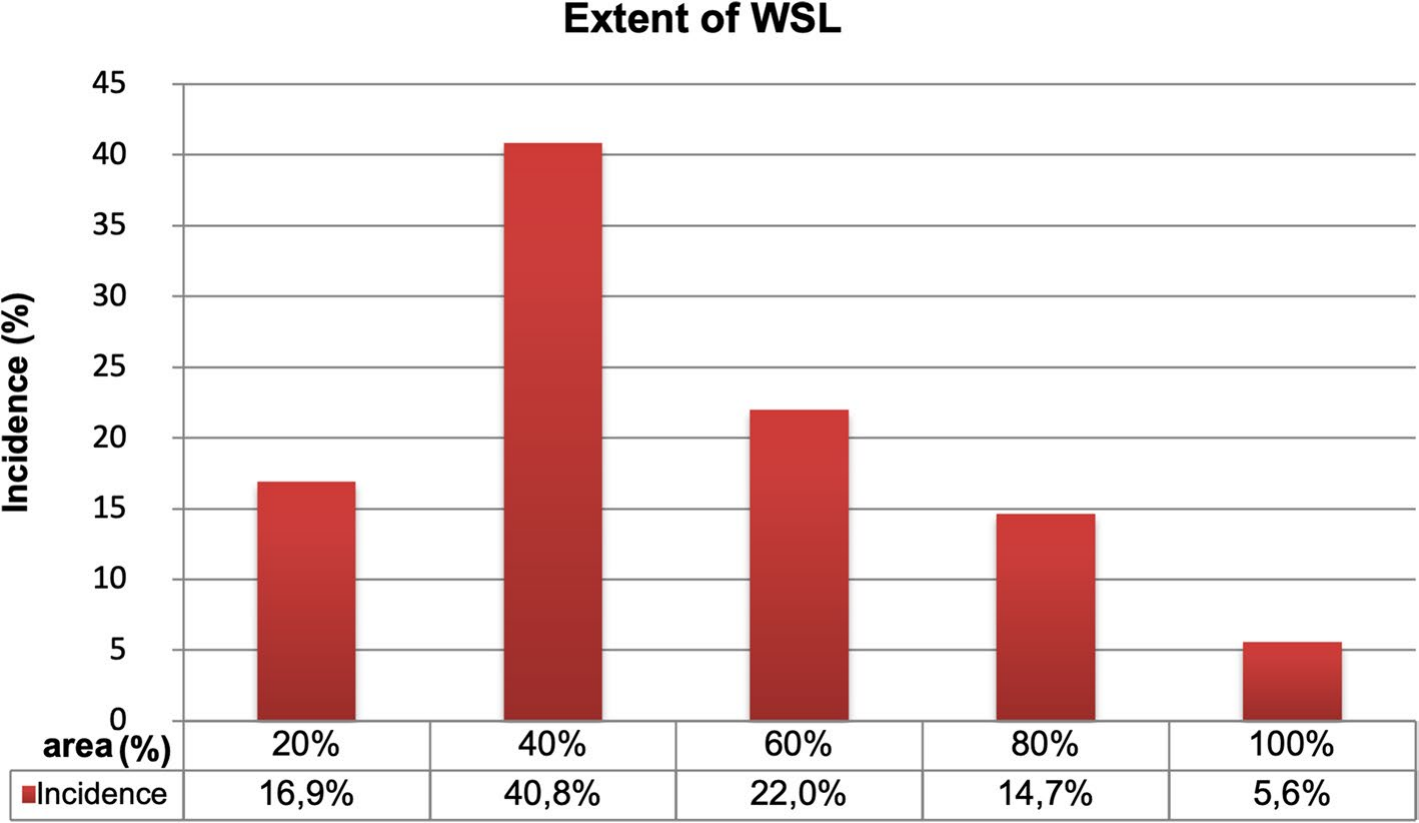
Distribution of the incidence according to extent (%).

16.9% of the newly affected tooth surfaces had enamel demineralizations on 20% of the surfaces. The majority (40.8%) of new cases exhibited enamel demineralizations on 40% of surfaces. 22% of tooth surfaces had an incidence with a severity of 60%. The lowest incidence could be detected with enamel demineralizations of 80% (14.6%) and 100% (5.6%) (Fig. 7).

### Logistic regression

The probability of WSL diagnosis prior to MB therapy is lower in female than male patients. The risk for female patients of developing a WSL is 0.89 times lower than for male patients. In this study we chose tooth 11 as a reference and teeth 12, 13, 14, 15, and 24 showed a statistically higher risk of contracting a WSL than tooth 11. The lower teeth and teeth 21, 22, 23, and 25 were found to be at a lower risk of contracting a WSL than the reference. Teeth 12, 13, 15, 21, 22, 23, 25, 34, 35, 44, and 45 (p > 0.05) showed a similar risk of developing a WSL before MB therapy as tooth 11. This first model was analyzed by factoring in the tooth’s surfaces as a parameter. Thereby, the risk of developing a WSL is statistically higher for the occlusal, distal and mesial surfaces than for the gingival area, with an increased risk factor of 1.33–1.61 (Table 3).

**Table 3:** Overview of the parameters for the logistic regression: WSL prior to MB therapy.

Parameter	Hypothesis testing		Odds ratio	95% Wald confidence interval for Exp(B)	
	df	Sig	Exp(B)	Lower value	Upper value
(Constant term)	1	0.017	0.063	0.006	0.608
Male			1		
Female	0.1	0.655	0.887	0.523	1.054
Tooth 11			1		
Tooth 12	1	0.329	1.202	0.831	1.739
Tooth 13	1	0.850	1.039	0.701	1.540
Tooth 14	1	0.033	1.569	1.036	2.377
Tooth 15	1	0.562	1.139	0.733	1.771
Tooth 21	1	0.377	0.894	0.696	1.147
Tooth 22	1	0.841	0.966	0.690	1.353
Tooth 23	1	0.760	0.936	0.611	1.433
Tooth 24	1	0.010	1.775	1.146	2.748
Tooth 25	1	0.968	0.989	0.578	1.693
Tooth 31	1	0.000	0.081	0.029	0.225
Tooth 32	1	0.000	0.177	0.089	0.351
Tooth 33	1	0.016	0.566	0.356	0.900
Tooth 34	1	0.580	0.869	0.529	1.428
Tooth 35	1	0.168	0.636	0.334	1.210
Tooth 41	1	0.000	0.075	0.030	0.184
Tooth 42	1	0.000	0.129	0.059	0.281
Tooth 43	1	0.000	0.311	0.174	0.555
Tooth 44	1	0.614	0.885	0.551	1.421
Tooth 45	1	0.293	0.740	0.422	1.298
Surface o	1	0.000	1.605	1.266	2.035
Surface d	1	0.001	1.325	1.115	1.574
Surface m	1	0.000	1.441	1.222	1.700
Surface g			1		
Age	1	0.134	1.110	0.968	1.273
Treatment time	1	0.972	0.996	0.778	1.275

The probability of a WSL diagnosis after MB therapy is lower in female than in male patients, as males have a 0.65 times greater risk than female patients.

In his case we again chose tooth 11 as a reference. Teeth 12, 13, 14, 15, 22, 23, 24, 25, 34, 35, 44 and 45 showed a statistically higher risk of developing a WSL than tooth 11. The upper and lower premolars (odds ratio = 1.51–2.52) were statistically conspicuous. The LJ anterior and canine teeth (31, 32, 41, 42) exhibited a lower risk of contracting a WSL than the reference tooth. Teeth 21, 33, and 43 showed a similar risk to tooth 11 (p > 0.05) of developing a WSL after MB therapy. The analysis based on the second model was concluded by factoring in the tooth surface as a parameter, just like the preceding analysis was. The occlusal, distal, and mesial surfaces were found to have a statistically higher risk of developing WSL than the gingival surfaces. As a result, the risk of WSL was increased by a factor of 1.24–1.37 times (Table 4).

**Table 4:** Overview of the parameters for logistic regression: WSL after MB therapy.

Parameter	Hypothesis testing		Odds ratio	95% Wald confidence interval for Exp(B)	
	df	Sig	Exp(B)	Lower value	Upper value
(Constant term)	1	0.945	0.938	0.153	5.764
Male			1		
Female	1	0.049	0.645	0.417	0.999
Tooth 11			1		
Tooth 12	1	0.000	1.846	1.388	2.456
Tooth 13	1	0.000	1.950	1.377	2.762
Tooth 14	1	0.000	2.379	1.657	3.414
Tooth 15	1	0.013	1.506	1.090	2.080
Tooth 21	1	0.291	1.126	0.903	1.403
Tooth 22	1	0.001	1.688	1.294	2.281
Tooth 23	1	0.000	2.474	1.771	3.455
Tooth 24	1	0.000	2.524	1.763	3.613
Tooth 25	1	0.000	2.005	1.383	2.907
Tooth 31	1	0.000	0.213	0.141	0.321
Tooth 32	1	0.000	0.387	0.265	0.567
Tooth 33	1	0.928	0.983	0.679	1.424
Tooth 34	1	0.000	2.161	1.464	3.189
Tooth 35	1	0.004	1.797	1.201	2.689
Tooth 41	1	0.000	0.226	0.146	0.350
Tooth 42	1	0.000	0.417	0.285	0.610
Tooth 43	1	0.771	1.061	0.712	1.581
Tooth 44	1	0.000	2.120	1.465	3.068
Tooth 45	1	0.000	2.042	1.370	3.046
Surface o	1	0.007	1.235	1.058	1.441
Surface d	1	0.000	1.372	1.218	1.547
Surface m	1	0.000	1.331	1.182	1.498
Surface g			1		
Age	1	0.582	1.033	0.921	1.159
Treatment time	1	0.133	0.878	0.741	1.040

The probability of diagnosing a new WSL after MB therapy is 0.7 times lower in female than in male patients.

In order to analyse the various tooth types, we again chose tooth 11 as a reference. Teeth 12, 13, 14, 22, 23, 24, 25, 34, 35, 43, 44 and 45 showed a statistically higher risk of developing a WSL than tooth 11. The upper and lower premolars (apart from 15) (odds ratio = 1.69–2.8) were found to be statistically conspicuous. The lower teeth (31 and 41) were at a lower risk of developing a WSL than the reference. Teeth 15, 21, 32, 33, and 42 showed a similar risk of developing a WSL after MB therapy as tooth 11 (p > 0.05).

As with the other analyses, we developed the third model analysis by factoring in tooth surfaces as a parameter. Mesial and gingival surfaces carried a similar statistical risk of developing a WSL, the risk for distal surfaces was slightly higher than for mesial and gingival surfaces and the occlusal area carried the lowest risk (Table 5).

**Table 5:** Overview of the parameters for the logistic regression: Incidence.

Parameter	Hypothesis testing		Odds ratio	95% Wald confidence interval for Exp(B)	
	df	Sig	Exp(B)	Upper value	Lower value
(Constant term)	1	0.430	0.938	0.153	5.764
Male			1		
Female	1	0.055	0.697	0.483	1.007
Tooth 11			1		
Tooth 12	1	0.003	1.730	1.210	2.475
Tooth 13	1	0.001	2.172	1.402	3.365
Tooth 14	1	0.027	1.688	1.060	2.688
Tooth 15	1	0.497	1.165	0.749	1.814
Tooth 21	1	0.100	1.287	0.953	1.739
Tooth 22	1	0.001	1.860	1.295	2.672
Tooth 23	1	0.000	2.474	1.771	3.455
Tooth 24	1	0.000	2.799	1.797	4.361
Tooth 25	1	0.012	1.851	1.147	2.986
Tooth 31	1	0.008	0.482	0.281	0.826
Tooth 32	1	0.336	0.783	0.476	1.289
Tooth 33	1	0.060	1.561	0.981	2.483
Tooth 34	1	0.000	2.796	1.697	4.608
Tooth 35	1	0.002	2.428	1.387	4.251
Tooth 41	1	0.009	0.469	0.266	0.829
Tooth 42	1	0.670	0.906	0.575	1.426
Tooth 43	1	0.001	2.313	1.437	3.721
Tooth 44	1	0.000	2.568	1.589	4.152
Tooth 45	1	0.000	2.725	1.552	4.787
Surface o	1	0.395	0.930	0.786	1.100
Surface d	1	0.022	1.157	1.021	1.310
Surface m	1	0.670	1.030	0.900	1.179
Surface g			1		
Age	1	0.055	0.911	0.828	1.002
Treatment time	1	0.095	0.876	0.750	1.023

## Discussion

For this retrospective study, digital, intraoral photgraphs of 121 patients (and therefore 9680 tooth surfaces) that received labial MB therapy as well as standardized oral hygiene instructions were analyzed. The study focused on upper and lower incisors, canine teeth, and premolars because their tooth surfaces are clearly visible on frontal as well as lateral photographs—despite the labially bonded brackets during MB therapy. Molars were not included in the analysis as the bands used on them impeded the evaluation of buccal tooth surfaces on lateral photographs. Benson et al. showed that the analysis of digital photographs is better suited for retrospectively evaluating WSL than analog photographs^34^. Accordingly, digital photographs allowed for a correct identification of demineralization cases 63% of the time, whereas analog photographs allowed for a 47% correct diagnosis rate. Previous studies of photographs have already emphasized that the evaluation of graphical material can vary due to differing exposure, saturations, and intensities^35^. This variability particularly affects the decision about whether a mild or severe WSL is presented. Nonetheless, healthy enamel can easily be differentiated from WSL^13^, which is also the reason why we conducted the analysis in a dark room. In terms of repeatability, we found that digital photographs allow for an easier repeated diagnosis of WSL than clinical inspection or a light microscopic study^36,37^. As a result, this method allows for effective documentation and analysis of the prevalence as well as severity of demineralization over a specific period of time^38^.

In order to develop a novel evaluation tool to increase the repeatability of the examination, so that the assessment could be performed by varying researchers or by the same researcher, a standardized procedure was used. The benefits of using the EDI are, that the division of the labial tooth surfaces around the bracket are divided into four areas, and that the scale is organized into percentages. Level 0 corresponds to an intact enamel surface, level 1 describes demineralization of less than 50% of the tooth surface, level 2 represents demineralization of more than 50% of the surface, and level 3 corresponds to demineralization of 100% of the inspected area or a carious lesion. AIt is difficult to compare the EDI with other analytical methods because it is not yet widely used^11^. The EDI evaluates the intensityof WSL but not the extent of them, meaning that eben a small cavitiation is categorized at the highest level (level 3). Hence, alongside intensity, the extent is of particular interest. To deliver more accurate conclusions on the extent of lesions on the tooth surface, we chose a digital graticule with concentric circles and an evaluation scale in increments of 20%. We considered the index by von der Fehr^39^ unfit for the retrospective examination of WSL with photographs as it is used for light microscopic studies of extracted teeth and their evaluation of the surface condition with regard to the opacity and, thus, the severity of WSL. This index, however, does not allow for conclusions concerning the extent of WSL, which is why it is only applied in in vivo examinations and in relation to other indices^2^.

The WSL index by Årtun and Brobakken^2^ however, considers the individual tooth surfaces surrounding the bracket and, thus, can sufficiently diagnose the extent of a WSL. They divided each vestibular tooth surface around the bracket into four areas: gingival, mesial, distal, and occlusal or rather incisal. They also modified the scale division: level 0 = no WSL, level 1 = WSL implies < 1/3 of the surface is uncovered by the bracket, level 2 = WSL implies > 1/3 of the surface is uncovered by the bracket, level 3 = WSL implies > 2/3 of the surface is uncovered by the bracket. Moreover, Årtun and Thylstrup^40^ combined their index with von der Fehr’s index to draw more profound conclusions on lesion severity.

In 1977, Cruzon and Spector devised another index for evaluating enamel opacity^41^. Mizrahi^12^ modified this index to draw conclusions on the severity of opacity, location, and dimension. However, this index only describes the dimension of WSL on the entire vestibular surface without generating a concise division of the areas surrounding the bracket.

The semi-quantitative index by Gorelick et al.^10^ is commonly used but similarly unfit for conducting a more detailed and site-specific analysis. It features four levels: level 1 = no WSL, level 2 = no lesion, mild demineralization, level 3 = no lesion, grave demineralization, level 4 = cavitation, presence of cavity. This WSL index by Gorelick et al. is suitable for depicting prevalence and incidence of demineralizations. The categorization of level 2 and 3 devolve, the index also does not divide the labial surfaces any further.

Contrary to the other methods we discussed earlier, the newly designed evaluation tool generates concise data on the extent of WSL. Other data useful for the critical evaluation of WSL, inter alia the so-called White Spot intensity and the WSL volume, are hereby not considered. Torlakovic et al.^42^ demonstrated that the whiter a WSL, the more it expanded. The WSL volume not only describes the extent of the lesion with respect to the surface but also considers the depth of penetration. Torlakovic et al.^42^ asserts that the traditional WSL index by Gorelick et al. is a weak indicator of the actual depth of demineralization. From this assertion we reason that, even if a WSL concerns an expansive surface, it does not automatically coincide with deeply penetrated enamel. The volume of WSL can only be determined on extracted teeth, and not in the clinical evaluation. Lesion depth can nonetheless be essential for performing potential WSL therapy.

The standardized procedure of this examination, which digitally adapts each photograph to the evaluation tool, allows for obtaining reproducible results regarding the extent of WSL. Other studies, which, above all, make use of the different refractive properties of healthy enamel and the demineralization body, have attempted to establish a correlation to the lesion’s depth. Thereby, polarized, fluorescent light sources and spectrophotometric measuring devices appear potentially well-suited for generating evident results regarding the formation of future WSL^36,42–50^. We found that the classification of WSL alongside an extension index, combined with a method that allows for conclusions on lesion depth, can constitutes as a useful tool for the diagnosis of labial WSL and ensuing therapeutic decisions.

In comparable studies, Mizrahi^12^ discovered a prevalence of 72.3% of WSL with examined patients prior to MB treatment; a study by Pancherz and Mühlich^18^ gave results of 70.4%. Enaia et al.^13^ and Årtun and Brobakken^2^ declared that 32.3% or 50.3% of the patients already had at least one previously damaged tooth prior to treatment. Merely Lovrov et al.^11^ found that only 2.5% of the examined patients in their cohort had a WSL level > 1. The cohort we examined accounted for a prevalence prior to MB treatment of 69.4%.

During MB treatment, we observed a continuous increase of WSL. 97.5% of our patients presented WSL on at least one tooth surface after MB treatment. A clinical study that classified WSL with quantitative light-induced fluorescence (QLF) calculated a prevalence of 97% after MB treatment^43^. Ogaard^24^ (96%), Mizrahi^12^ (84%), Enaia et al.^13^ (73.5%), and Banks and Richmond^22^ (73%) came to results that were equally high. In contrast, Gorelick et al.^10^, Geiger et al.^21^, and Lovrov et al.^11^ discovered that 49.6%, 33.8%, 32.3%, and 22.6% of the patients respectively had a WSL after MB therapy. The large variance of the occurrence of WSL between the studies mentioned above mainly derives from differences in their analytical approach^15^. The above mentioned analyses detected the degree of demineralization above all by using visual-clinical inspection. Boersma et al.^43^ discovered that all visually detected WSL were also correctly identified by using the QLF method. Furthermore, this method turned out to be more sensitive than visual examination when it came to an early diagnosis of WSL. Thus, the light-induced fluorescence (QLF) detected more WSL than the visual method. However, applying the QLF method is far more sophisticated and the design of the study must beconsidered as a further influencing factor. Retrospective examinations offer the advantage that a patient population can be re-examined quickly. The results also represent the emergence of WSL that appeared in the orthodontic practice with routine treatment^13^. Data such as social origin, additional fluoridations, or the duration and regularity of MB treatment have not been included in the evaluation. On the basis of prospective examinations, we can further evaluate other factors, and exclude patients from the study with bad or improvable oral hygiene or of patients with an already existing WSL^33^. Another observation is the so-called Hawthorne effect, which implies that a patient’s self-motivation considerably increases by participating in a study. This may ultimately have positive implications on their oral hygiene^51^.

Another essential difference that may explain the variance between the studies is the selection of the teeth subject to examination. Similar to our study, the majority of researchers analyzed the UJ and LJ anterior and canine teeth as well as the first and second premolars that were provided with a bracket^2,12,18,22^. Lucchese et al.^19^, Lovrov et al.^11^, and Ogaard^24^, however, also examined the banded molars. The inclusion of canine teeth, premolars, and molars significantly increases the probability to of diagnosing a WSL. As a result, the percentaged data of WSL prevalence cannot be compared to other studies such as Enaia et al.^13^, which solely analyzed the upper anterior teeth. Moreover, methodological variations occur on account of the use of different WSL indices, bracket and bonding matierals, oral hygiene measures, and treatment durations. However, the broad age range among patients and, potentially, their varying ethnic backgrounds can similarly result in these variations^52,53^.

In our patient population, 74.1% of female patients presented a WSL prior to and 96.5% after MB therapy. In comparison, we diagnosed at least one tooth with a WSL for 65.1% of male patients prior to and 98.4% after MB therapy. One plausible cause for the higher number of female patients with WSL at the beginning of the treatment is most likely a past tooth eruption. The affected tooth is exposed to an oral cavity for a longer period and thus bears a higher risk of developing a WSL for patients of the same age group^18^. The gender of the patient is statistically insignificant to the development of a WSL prior to MB therapy. However, after MB therapy, the difference is statistically conspicuous (p = 0.049), as more male than female patients present a WSL. The logistic regression of this study thereby refers to all tooth surfaces. Therefore, we conclude that at any point in time, more tooth surfaces of males than females have been affected, even though fewer male patients presented a WSL. These results also correspond to the findings of Mizhari^12^ and Zachrisson and Zachrisson^54^ who observed worse oral hygiene or compliance with male than with coeval female patients during orthodontic treatment.

The evaluation of WSL prevalence prior to MB therapy with respect to each individual tooth showed a symmetric distribution of WSL on the different quadrants of the same jaw. The first and second premolars of UJ and LJ were more strongly affected than canine teeth or lateral incisors of the same jaw. The central incisors exhibited the lowest prevalence both prior to and after MB treatment. For teeth 14 and 24, the binary logistic regression resulted in a statistically increased risk of developing a WSL prior to MB therapy. In comparison, the lowest risk was calculated for the LJ anterior and canine teeth and teeth 14 and 24 remained having the highest risk of developing WSL after MB therapy. Furthermore, there is a statistically increased risk of developing a WSL for teeth 12, 22, 13, 23, 15 and 25, as well as for the first and second premolars of the LJ. These results correspond to the findings of other studies for the lower teeth^2,10,19,21,24^. The anatomic proximity to the excretory duct of the salivary glands of the glandula submandibularis as wells as sublingualis seems to be mainly responsible for the low prevalence in the lower anterior teeth^55^. In the evaluation by Pancherz and Mühlich^18^ an accumulation of changes in the enamel on the upper central and lateral incisors prior to MB treatment was discovered. A past eruption on the remaining upper anterior teeth and the resulting longer exposure to the oral cavity^18,56^ were stated as a possible cause for these changes. Moreover, they also diagnosed an increase of demineralization on the lateral incisors after MB treatment^2,10,12,21,54^. The lateral incisor seems to be particularly predisposed due to its smaller clinical crowns, and the pasting of the bracket leaves only a small space between the bracket and the gingival margin. This area is hardly accessible for oral hygiene and thus entails a higher demineralization risk. The results of our study are also confirmed by the results found by Lovrov et al.^11^ who proved that in the upper jaw, the first premolars have the highest prevalence of WSL both before and after MB treatment. Tufekci et al.^57^ examined the prevalence of WSL after wearing the MB appliance for 6 and for 12 months and could not detect a difference between the various tooth types. An evaluation is difficult with the present MB appliance because it does not allow for an accurate identification of labial WSL^52^.

A final evaluation of WSL prevalence allowed for an analysis of the different tooth surfaces surrounding the bracket. The risk of developing a WSL before MB treatment was statistically higher for occlusal, distal, and mesial surfaces in comparison to gingival tooth surfaces. After MB therapy, the distal surface is more often affected than mesial, occlusal, and gingival surfaces. Cariology established that the highest susceptibility to cavities is on the approximal tooth surfaces, which more often concerns the distal than the mesial surface^58^. Comparable orthodontic studies attributed the highest risk of contracting a WSL to gingival tooth surfaces^10,54^. The gingival area is normally the smallest tooth surface, and therefore facilitates plaque retention^2,22^. Like our own patient population, Robertson et al.^25^ describes a tendency to incisal and occlusal demineralizations. When compared to literature, this appears to be a nontypical distribution, though it is not a systematic mistake. Firstly, Robertson et al.’s^25^ results were collected by two independent researchers; secondly, there was a high level of reliability between examiners. It can therefore be concluded that WSL prevalence in both patient populations was focused on the incisal quadrants. WSL incidence in our patient population amounted to 29%. This means that 29% of the patients who had no WSL prior to MB treatment, exhibited demineralization after MB therapy on at least one tooth surface. Compared to the incidence discussed in the literature, this constitutes a relatively small number of newly affected tooth surfaces. The analysis by Banks and Richmond^22^ shows a considerably higher incidence: 74% of the patients had a WSL after MB treatment for the first time. 62% of the patient population from Pancherz and Mühlich^18^, were diagnosed with new or enlarged enamel lesions. In Enaia et al.’s^13^ retrospective study, 60.9% of the examined upper anterior teeth had a WSL after MB therapy. Gorelick et al.^10^ examined 121 patients in total and thereby detected an incidence of 49.6% and only Chapman et al.’s^15^ study showed a lower incidence (36%). The Chapman et al. study only examined the digital photographs of the upper teeth 14–24. The studies present a wide variability of results, as the evaluation of prevalence prior to and after MB treatment did. The analytic approach, the number and type of the examined teeth, and the WSL index used for diagnosis only allowed for a limited comparison of the WSL incidence in the studies at hand.

In this study, 23.2% of the female patients presented a WSL for the first time on at least one tooth after MB therapy whereas the incidence in male patients was 33.9%. We considered this difference statistically insignificant. We acknowledged that this result did not correspond to Gorelick et al.’s^10^ conclusions (incidence of 44% for male and of 55% for female patients). However, it is important to note that Gorelick et al.’s^10^ patient population consisted of 49 boys and 72 girls, which did in fact impact their results. Ogaard^24^ also did not detect a statistically significant gender variation. On the other hand, Boersma et al.^43^ discovered a statistically significant difference between the incidence of male (40%) and female (22%) patients.

The evaluation of new post-therapy WSL on different types of teeth in the UJ was able to conclude that canine teeth and premolars had the highest WSL incidence (71–79%). 61–63% of cases were diagnosed with a new labial WSL on the lateral incisors of the UJ, on at least one tooth surface. In the LJ, the first and second premolars are most often affected by WSL (64–76%), though the premolars (aside from 15) of the UJ and LJ are statistically conspicuous. Similar to the prevalence, the results of the UJ here diverge from those discussed in the literature. In their study, Banks and Richmond^22^ detected the highest susceptibility to WSL for the lateral incisors and the canine teeth of the UJ. They also implied that using so-called “solder arch hooks” (hooks soldered on the archwire) complicates oral hygiene and contributes to an increase in WSL developement. This finding also corresponds to the results of other studies and allows for the conclusion that small tooth surfaces between the bracket and gingival margin increase the accumulation of plaque and food remains, inturn leading to greater risk of demineralization^2,10,21^. The age of the patients at the beginning of MB treatment also proved to be an impacting factor in the development of new WSL in the UJ anterior teeth area^59^. Starting therapy at a pre-pubertal age (9–12 years) was associated with a higher WSL risk^15^.

It can be cocluded that the development of new WSL are symmetrically distributed on the left and right sides of the jaw. The studies at hand thereby correspond to the results of this study with respect to the WSL incidence in the UJ as well as the LJ premolars and canine teeth.

The high disposition to WSL of the lateral incisor could not be proven in this study. One reason for this might be the particular approach of acknowledging this risk and amending oral hygiene instructions accordingly, which can result in the reduction of plaque retention in this area. Additional retention sited were created with the use of hooks or loops between canine teeth and lateral incisors created additional retention sites. We only used these tools with 7 out of 121 patients in our cohort, thus the risk of demineralization on the lateral incisors was lower.

Finally, we analyzed the various tooth surfaces around the bracket in order to determine the incidence. The logistic regression analysis concluded in a statistically increased risk of developing a new WSL for the distal tooth surface. For the mesial and gingival surfaces, the risk of diagnosing a new WSL during MB treatment is equally high, while the occlusal surface bears the lowest risk. Epidemiological studies on caries incidence are able to prove that the risk of diagnosing a new cavity is higher on approximal tooth surfaces, regardless of an MB appliance^58^. The result of this study suggests that the presence of an MB appliance results in an increased tendency for developing a new WSL on the gingival tooth surface^25^. Nevertheless, this hypothesis must be considered with reservations as it can only be confirmed through a control group without an MB appliance. Banks and Richmond^22^ concluded that almost 2/3 of the newly developed WSL were located in the gingival tooth area. The small area between the gingival margin and the bracket thus present an increased risk of plaque retention. The knowledge of these risk factors influences the orthodontists’ approach to the adhesive bonding of brackets and oral hygiene instructions. Consequently, a reduction of the effects of the MB appliance on the development of new WSL is to be expected.

In order to generate a large sample population and exclude any possible bias, like the Hawthorne study shows, this retrospective study design was chosen to resemble daily clinical practice routines^51^. However, the digital photographs were analyzed by only one examiner, which presented a limitation to the study. Future studies should focus on more observers and include intra-observer agreement studies.

## Conclusion

Based on photographic records from standardized orthodontic treatment, results of this study showed that patients undergoing fixed orthodontic treatment have a significantly high risk of developing new WSL during orthodontic treatment. Clinicians should consider poor oral hygiene to be the main risk factor in the development of WSLs during fixed orthodontic treatment. Labial MB treatment within an adolescent population induces WSL particularly on canine teeth and premolars. The extent of labial WSL was evaluated concisely based on a high intra-rater reliability on our specially developed evaluation index (digital graticule with concentric circles). Nonetheless, further studies with more examiners, and inter-examiner agreement studies are required to evaluate the validity and reproducibility of the novel developed digital evaluation tool.

## Data Availability

The data used for analysis has been referenced in the text or tables of the paper.

## Abbreviations

WSL: White spot lesions
MB: Multibracket appliance
EDI: Enamel decalcification index
UJ: Upper
LJ: Lower jaws
TS: Tooth surfaces
QLF: Quantitative light-induced fluorescence
g: Gingival
m: Mesial
d: Distal
o: Occlusal
ICC: Intraclass correlation coefficient
